# Supplementary material for: In silico cancer immunotherapy trials uncover the consequences of therapy-specific response patterns for clinical trial design and outcome
Source: Nat Commun. 2023 Apr 24;14:2348. doi: 10.1038/s41467-023-37933-8 (PMC10125995; doi:10.1038/s41467-023-37933-8)
Supplement: Supplementary file 1 — Supplementary Information [file 41467_2023_37933_MOESM1_ESM.pdf]

In silico cancer immunotherapy trials uncover the consequences of therapy-specific response patterns for clinical trial design and outcome

Supplementary Information

Jeroen H. A. Creemers[1,2], Ankur Ankan[3], Kit C. B. Roes[4], Gijs Schröder[3], Niven Mehra[5], Carl G. Figdor[1,2], I. Jolanda M. de Vries[1], Johannes Textor[1,3#]

[1] Department of Tumor Immunology, Radboud Institute for Molecular Life Sciences, Radboud University Medical Center, Nijmegen, The Netherlands

[2] Oncode Institute, Nijmegen, The Netherlands

[3] Data Science group, Institute for Computing and Information Sciences, Radboud University, Nijmegen, The Netherlands

[4] Department of Health Evidence, Section Biostatistics, Radboudumc, Nijmegen, The Netherlands

[5] Department of Medical Oncology, Radboudumc, Nijmegen, The Netherlands

Corresponding author email address:
[#]johannes.textor@ru.nl

Supplementary Figures

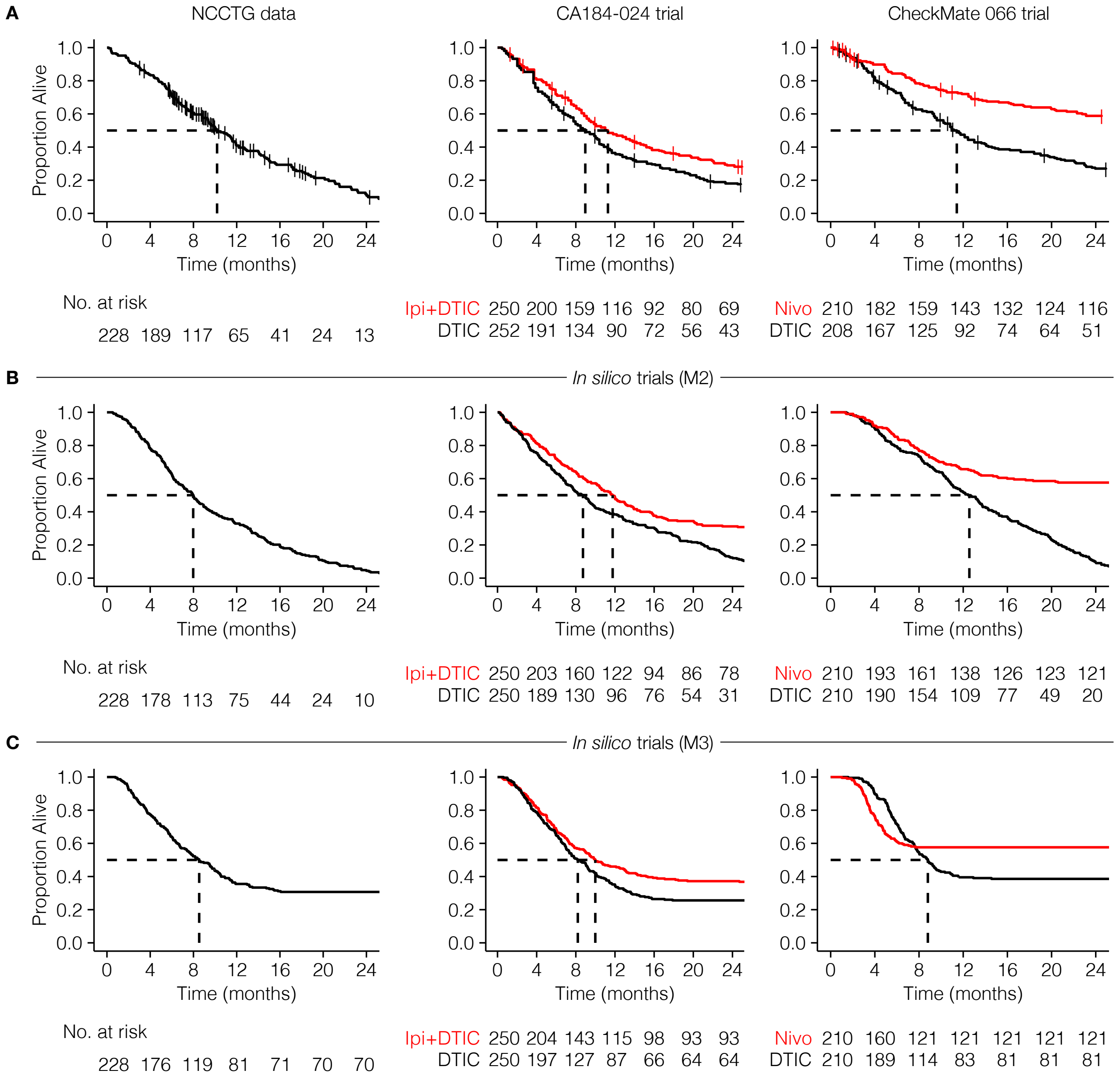

Supplementary Figure 1: **Fits of models M2 and M3 to the clinical trial data.** (A) Clinical trial data (identical to Figure 3A in the main manuscript and displayed for visual comparison). (B) Fits of model M2. (C) Fits of model M3.

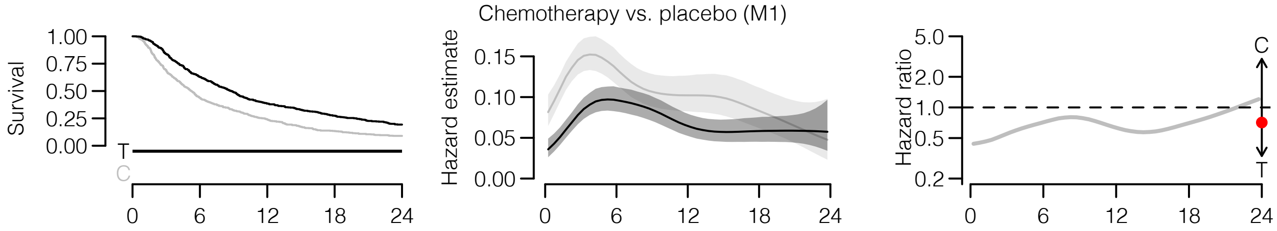

Supplementary Figure 2: ***In silico* chemotherapy trial that approximately fulfills the proportional hazards assumption.** Lines and shading in middle column: estimated hazards and 95% CIs (see Methods); n=600 simulated patients per arm.

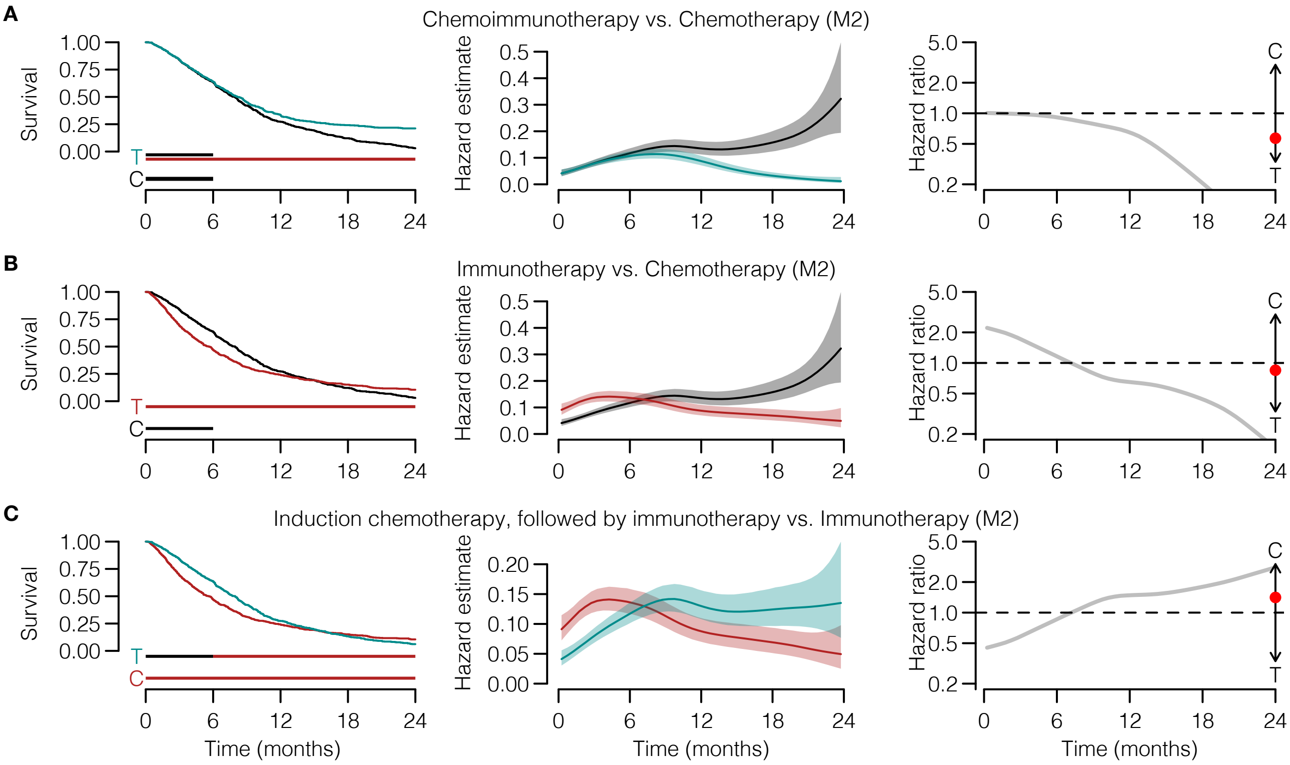

Supplementary Figure 3: **Predicted immunotherapy trial survival curves for model M2** based on its fit to the CA184-024 trial data. (A) Chemoimmunotherapy versus chemotherapy; (B) immunotherapy versus chemotherapy; (C) induction chemotherapy, followed by immunotherapy versus immunotherapy. Lines and shading in middle column: estimated hazards and 95% CIs (see Methods); n=600 simulated patients per arm.

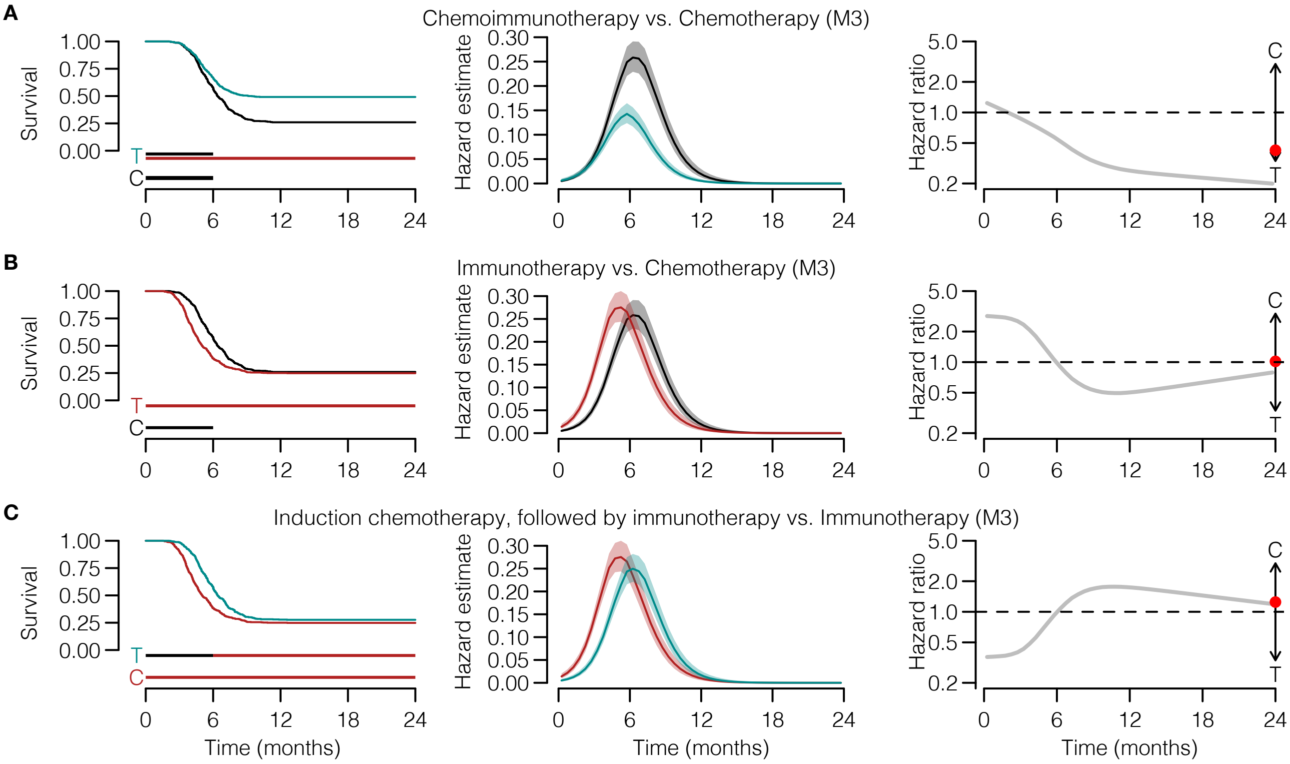

Supplementary Figure 4: **Predicted immunotherapy trial survival curves for model M3** based on its fit to the CA184-024 trial data. (A) Chemoimmunotherapy versus chemotherapy; (B) immunotherapy versus chemotherapy; (C) induction chemotherapy, followed by immunotherapy versus immunotherapy. Lines and shading in middle column: estimated hazards and 95% CIs (see Methods); n=600 simulated patients per arm.

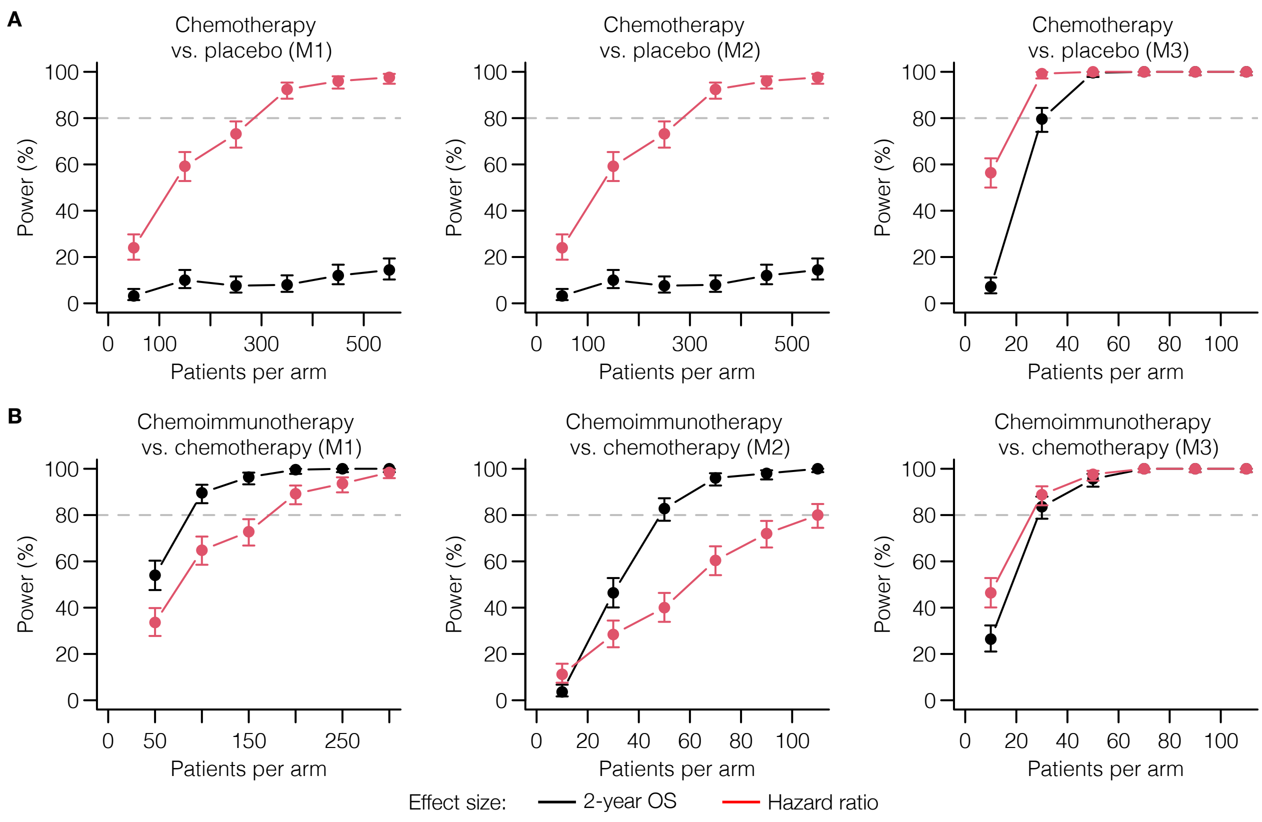

Supplementary Figure 5: **Power analyses using simulated trials.** (A) Chemotherapy versus placebo; (B) chemoimmunotherapy versus immunotherapy. Points and error bars: estimated proportions and 95% CIs from a binomial test; n=250 simulated trials per sample size.

R package	Reference	Version	Source
bshazard	[1]	1.1	CRAN
dplyr	[2]	1.0.10	CRAN
ggplot2	[3]	3.3.6	CRAN
ggsankey	[4]	0.0.99999	github.com/davidsjoberg/ggsankey
grid	[5]	4.2.0	part of R 4.2.0
IPDfromKM	[6]	0.1.10	CRAN
ks	[7]	1.13.5	CRAN
ldbounds	[8]	2.0.0	CRAN
Rccp	[9]	1.0.9	CRAN
survival	[10]	3.3-1	CRAN
survminer	[11]	0.4.9	CRAN

Supplementary Table 1: R packages used in this manuscript with references and versions.

Supplementary References

[1] Paola Rebora, A. S. & Reilly, M. *bshazard: Nonparametric Smoothing of the Hazard Function* (2018). URL https://CRAN.R-project.org/package=bshazard. R package version 1.1.

[2] Wickham, H., François, R., Henry, L. & Müller, K. *dplyr: A Grammar of Data Manipulation* (2022). URL https://CRAN.R-project.org/package=dplyr. R package version 1.0.10.

[3] Wickham, H. *ggplot2: Elegant Graphics for Data Analysis* (Springer-Verlag New York, 2016). URL https://ggplot2.tidyverse.org.

[4] Sjoberg, D. *ggsankey: Sankey, Alluvial and Sankey Bump Plots* (2022). URL https://github.com/davidsjoberg/ggsankey. R package version 0.0.99999.

[5] R Core Team. *R: A Language and Environment for Statistical Computing*. R Foundation for Statistical Computing, Vienna, Austria (2022). URL https://www.R-project.org/.

[6] Liu, N. & Lee, J. *IPDfromKM: Map Digitized Survival Curves Back to Individual Patient Data* (2020). URL https://CRAN.R-project.org/package=IPDfromKM. R package version 0.1.10.

[7] Duong, T. *ks: Kernel Smoothing* (2022). URL https://CRAN.R-project.org/package=ks. R package version 1.13.5.

[8] Casper, C., Cook, T. & Perez., O. A. *ldbounds: Lan-DeMets Method for Group Sequential Boundaries* (2022). URL https://CRAN.R-project.org/package=ldbounds. R package version 2.0.0. Based on FORTRAN program ld98.

[9] Eddelbuettel, D. & François, R. Rcpp: Seamless R and C++ integration. *Journal of Statistical Software* **40**, 1–18 (2011).

[10] Terry M. Therneau & Patricia M. Grambsch. *Modeling Survival Data: Extending the Cox Model* (Springer, New York, 2000).

[11] Kassambara, A., Kosinski, M. & Biecek, P. *survminer: Drawing Survival Curves using 'ggplot2'* (2021). URL https://CRAN.R-project.org/package=survminer. R package version 0.4.9.
